# Correction: Dynamic conformational equilibria in the active states of KRAS and NRAS

**DOI:** 10.1039/d6cb90018f

**Published:** 2026-05-05

**Authors:** Enrico Rennella, Chrystèle Henry, Callum J. Dickson, Florian Georgescauld, Thomas E. Wales, Dirk Erdmann, Simona Cotesta, Michel Maira, Richard Sedrani, Saskia M. Brachmann, Nils Ostermann, John R. Engen, Lewis E. Kay, Kim S. Beyer, Rainer Wilcken, Wolfgang Jahnke

**Affiliations:** a Novartis Biomedical Research Basel Switzerland wolfgang.jahnke@novartis.com rwilcken@flaretx.com kim.beyer@novartis.com; b Department of Biochemistry, University of Toronto Toronto Canada; c Novartis Biomedical Research Cambridge MA USA; d Department of Chemistry and Chemical Biology, Northeastern University Boston MA USA

## Abstract

Correction for ‘Dynamic conformational equilibria in the active states of KRAS and NRAS’ by Enrico Rennella *et al.*, *RSC Chem. Biol.*, 2025, **6**, 106–118, https://doi.org/10.1039/D4CB00233D.

The authors regret two instances of incorrectly displayed data in the published article whereby the conformation states corresponding to inactive (state 1) and active (state 2) have been mislabelled and should be swapped.

One instance is in a line of text on page 113 under the subheading “Hydrogen/deuterium exchange mass spectrometry” and a second instance is in Fig. 2 in the originating manuscript. The corrected manuscript text, figure and accompanying text are below. The conclusions of the paper have not been affected.

**Fig. 2:**
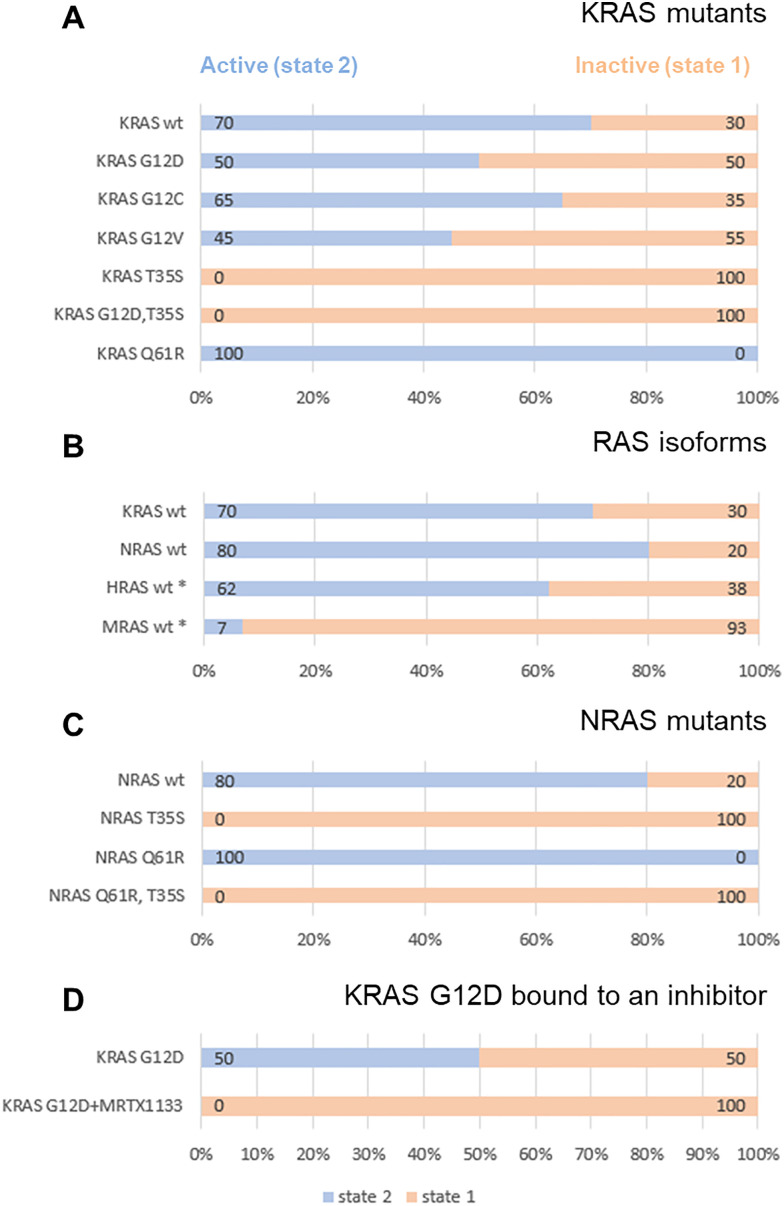
Populations of state-1 (peach) and state-2 (blue) as determined by ^31^P NMR in (A) KRAS mutants, (B) RAS isoforms, (C) NRAS mutants, (D) KRAS G12D complexed with an inhibitor. All corresponding ^31^P spectra are shown in Fig. 1A. All RAS proteins were bound to GMPPNP and measurements were carried out at 7 °C. The asterisk (*) refers to previously published data on HRAS^22^ and MRAS.^25,29^.

“Again, this is in line with the observations from ^31^P NMR, although the extent to which these regions saw increases in deuterium incorporation in HDX-MS was surprisingly large, given the relatively small shift seen in ^31^P NMR from active state-2 to inactive state-1 (70/30 in wildtype to 50/50 in G12D).”

The Royal Society of Chemistry apologises for these errors and any consequent inconvenience to authors and readers.

